# Productive and Qualitative Traits of Amaranthus Cruentus L.: An Unconventional Healthy Ingredient in Animal Feed

**DOI:** 10.3390/ani10081428

**Published:** 2020-08-14

**Authors:** Fabio Gresta, Giorgia Meineri, Marianna Oteri, Carmelo Santonoceto, Vittorio Lo Presti, Annalisa Costale, Biagina Chiofalo

**Affiliations:** 1 Department of Veterinary Sciences, University of Messina, 98168 Messina, Italy; fgresta@unime.it (F.G.); vittorio.lopresti@unime.it (V.L.P.); 2 Department of Veterinary Sciences, University of Turin, 10095 Grugliasco, Italy; giorgia.meineri@unito.it; 3 Department of Chemical, Biological, Pharmaceutical and Environmental Sciences, University of Messina, 98168 Messina, Italy; moteri@unime.it; 4 Department AGRARIA, University Mediterranea of Reggio Calabria, 89124 Reggio Calabria, Italy; csantonoceto@unirc.it; 5 Department of Drug Science and Technology, University of Turin, 10125 Torino, Italy; annalisa.costale@unito.it

**Keywords:** amaranth, germplasm, agronomic traits, oil composition, phenolic compounds, antioxidant activity, feedstuff

## Abstract

**Simple Summary:**

*Amaranthus cruentus* (red amaranth) can be considered a very interesting crop for its high nutritional and functional value. In this study, agronomic traits, oil content, fatty acid composition, quality indices, antioxidant activity, and total phenolic compounds were studied on eight *A. cruentus* accessions grown in Southern Italy. Data showed a valuable seed yield (0.27 kg/m^2^, on average) comparable to the main cereals used for animal feeding, a higher oil content compared to that of conventional cereals such as maize with a fatty acid profile composed approximately for the 60% of unsaturated fatty acids, and a valuable antioxidant potential and phenolic compounds that are far superior to corn and wheat. The studied seed-oil composition revealed a high content of essential fatty acid n-6 (linoleic acid) and n-9 (oleic acid). Therefore, this species can be used as pseudo-cereals to balance the animal diet according to animal species requirements and to the different metabolic pathways of unsaturated fatty acids in ruminants and monogastrics. In conclusion, *A. cruentus* may be of potential value as an oilseed crop of importance to areas of the Mediterranean and a high-quality alternative feed ingredient to traditional cereal grains.

**Abstract:**

Agronomic traits, oil content, fatty acid composition, antioxidant activity, and total phenolic content were studied on eight *A. cruentus* accessions cultivated in Southern Italy. A one-way ANOVA model was performed to compare accessions and the Principal Components Analysis was applied to identify patterns in our dataset and highlight similarities and differences. *A. cruentus* showed valuable seed yield (0.27 kg/m^2^, on average) comparable to the main tradition cereals used for animal feeding. Seed-oil composition showed significant differences among the accessions. Data showed a higher lipid content than most cereal grains (from 5.6 to 7.3%). Approximately 60% of fatty acids were unsaturated; linoleic fatty acid ranged from 19 to 34%, oleic acid from 29 to 36%, and alfa-linolenic fatty acid from 0.3 to 0.5%, respectively. The saturated/unsaturated fatty acid ratio ranged from 0.5 to 0.8, the hypocholesterolemic:hypercholesterolaemic ratio from 1.7 to 2.7, the Atherogenic Index from 0.38 to 0.66, the Thrombogenic Index from 0.85 to 1.48, the total phenolic content from 0.14 to 0.36 mg/g seeds, and the antioxidant activity (DPPH^•^) from 0.30 to 0.50. The studied seed-oil composition evidenced *A. cruentus* as a healthy ingredient for animal feed and consequently, as a possible substitute for traditional cereals. Accessions from Mexico and Arizona emerged for their high qualitative traits.

## 1. Introduction

*Amaranthus cruentus* (red amaranth) is an invasive, fast-growing weed originating in Central-South America [1]. It is one of about 70 species of the Amaranthaceae family [2] and was probably domesticated from *A. hybridus* [3]. *A. cruentus* is a drought-resistant annual, erected, broad-leaf plant, with great colored inflorescences producing small cereal-like edible grains. Each plant of *A. cruentus* can produce millions of seeds as a strategic adaptive germination pattern [4], which allows them wide dissemination and rapid colonization of new environments. *A. cruentus*, along with other species of the same family, was also an important ingredient of the diet of the pre-Columbian populations [1]. Still today, in Mexico, the grain of *A. cruentus is* used to produce a snack popped candy named “Alegria” [5]. *A. cruentus* is the most important Amaranth species cultivated in Africa both for the leaf as a vegetable and for the grain, showing high grain production potentiality [6]. It is also well adapted to a hot climate since Amaranth species, as well as corn and sorghum, belong to a class of plants with a C4-photosynthetic pathway able to maximize photosynthetic efficiency in high light intensity conditions and high temperatures. On the other hand, they are injured by temperatures below 4 °C [1]. Moreover, *A. cruentus* has high nutritional and functional values [7]: It shows, among others, an interesting fatty acid profile and a valuable antioxidant potential [8] and phenolic compounds, well-known as antioxidants. Conforti et al. [9] showed that amaranth leaf extracts contained phenolics, inhibited nitric oxide production, and scavenged free radicals. The reducing potential and antioxidant activity in lipid systems of various parts of the amaranth shoot system were determined as well [10].

Lipid seems to be interesting not only for its quantity but also for its fatty acid profile characterized by three dominant fatty acids: Palmitic, oleic, and linoleic acids; the oil is highly unsaturated, containing more than 70% unsaturated fatty acids [11]. For these reasons, *A. cruentus* is gaining importance among pseudo-cereal grain as an unconventional grain crop suitable not only as food but also as animal feed [12,13,14,15,16], as a possible substitute of traditional cereals for the health food market.

In livestock nutrition, *Amaranthus* can be used in different ways, as a grain and as fresh, dried, or ensiled forage [13,17,18]. The utilization of amaranth grain is particularly interesting after the occurrence of bovine spongiform encephalopathy and consecutive prohibition of meat-and-bone meals in the nutrition of all farm animal species in Europe [19]. The results on feed intake, feed conversion, and live weight gains varied considerably, within-species differences in animal responses due to natural variation and age, feed formulation, and processing method used [20,21,22,23,24,25].

Studies have demonstrated that this plant could be considered as a nutrient substitute for conventional feed for rabbits [26], pigs [24,27], chickens [28], and ruminants [29], especially in tropical and sub-tropical regions.

Notwithstanding these great potentialities, *A. cruentus* is still a neglected and underutilized crop, and information on the yield and fatty acid profile of this species, above all in a Mediterranean environment, is slender and fragmentary. To contribute to its diffusion, it does need to identify accessions with profitable yield and high-quality traits of the grain, able to compete with other grain crops.

With this in mind, the present research aims to explore the agronomic traits, oil, fatty acid content, antioxidant activity, and total phenolic content of the eight accessions of *A. cruentus*.

## 2. Materials and Methods

### 2.1. Field Experiment and Plant Material

The trial was carried out in 2014 in Bovalino (RC) (20 m a.s.l. 38°08′ N, 16°10′ E) in the Jonic coast of Calabria (Southern Italy) on 8 Amaranth accessions obtained from the USDA (Washington, DC, USA), seed bank.

Seeds were previously grown in a nursery and then transplanted in plots. Sowing was performed manually on the 21st March inside a nursery in expanded polystyrene trays and kept at 26 °C and 85% (±5%) RH. On the 23rd March, seedlings were moved to a greenhouse (19 °C ± 4 °C) and on the 25th April, plants with 4 true leaves were transplanted in a sandy-loam soil with a low amount of nitrogen (0.6 g/kg) and a high amount of assimilable phosphorus (80 mg/kg P_2_O_5_) and exchangeable potassium (241 mg/kg K_2_O). A density of 10 plants of m^2^ was adopted, 1.0 m apart with an intra-row distance of 0.10 m. A randomized block design with 3 replications was adopted with an elementary plot of 9 m^2^ (3 × 3 m). Seed density and plot dimensions were forced by the exiguous amount of seed received. The soil was cultivated with wheat in the previous year and was then shallow plowed and fertilized with 40 kg/ha of N, 80 kg/ha of P_2_O_5_, and 60 kg/ha of K_2_O before transplant. Just before anthesis, a further supply of 80 kg/ha of N as ammonium nitrate was broadcasted. Water supplies were assured with a drip system supplying a total volume of 3,200 m^3^/ha. Harvest was carried out from 28 June to 5 July in relation to the degree of maturation of the different accessions, and seeds were threshed with a laboratory thresher. Weeds were managed by hand.

During the trial, the main meteorological traits were recorded by a data logger placed next to the experimental field.

The average temperature ranged from 17.7 °C at the end of April to 25.2 °C at the end of June. The lowest temperature was recorded in April at sowing (14.6 °C), while the highest was recorded at the end of June (30.1 °C). As usual, in summer in the Mediterranean climate, rainfall during were inconsistent (26.4 mm).

### 2.2. Productive and Qualitative Measurements 

For each replication, at the end of the crop cycle, in agreement with the maturation of the different accessions, plant height was measured on 10 plants, and 1000 seeds weight, and seed yield were determined on a sample area of 2 × 2 m in the centre of the plot.

*A. cruentus* grains were analyzed by the GC–FID for the determination of the fatty acid methyl esters (FAME). Lipids were extracted according to the method described by Folch et al. [30]. The fatty acids methyl esters (FAMEs) were prepared by using a solution of sulfuric acid/methanol (1:9, *v*/*v*) [31].

For FAMEs, a gas chromatograph with a FID detector (Agilent Technologies 6890 N, Palo Alto, CA, USA) equipped with an Omegawax 250 column (Supelco, Bellefonte, PA, USA; 30 m × 0.25 mm i.d., 0.25 μm film thickness) was used. The column temperature was programmed: An initial isotherm of 100 °C (5 min), an increment of 4 °C/min, and a final isotherm of 240 °C (20 min). The temperature of the injector and detector was 250 °C. The injection volume was 0.5 μL. The carrier gas used was helium (1 mL/min), and the split ratio was 1:50.

Fatty acids were identified by comparing the relative retention times of FAME peaks from samples containing standards from Supelco (mix 37 FAMEs, Supelco, Bellefonte, PA, USA). Chromatogram peak areas were acquired and calculated using a Chemstation software (Agilent, Santa Clara, CA, USA). The concentration of each fatty acid was expressed as g/100 g, considering 100 g as the summation of the areas of all FAMEs identified. For each sample, the chromatographic analysis was replicated 3 times.

The amount of each fatty acid was used to calculate the indices of atherogenicity (AI) and thrombogenicity (TI), as proposed by [32], the hypocholesterolemic:hypercholesterolaemic ratio (HH), as suggested by Santos-Silva et al. [33], and the peroxidation index (PI) as proposed by Luciano et al. [34]:

$$AI=\frac{C12:0\;+\;4\;\left(\;C14:\;0\right)\;+\;C16:0}{\Sigma \;\mathrm{MUFA}\;+\;\Sigma \;n-6\mathrm{PUFA}\;+\;\Sigma \;n-3\mathrm{PUFA}}\;TI=\frac{C14:0\;+\;C16:0\;+\;C18:0}{\;\left(\;0.5\;\times \;\Sigma \;\mathrm{MUFA}\right)+\left(0.5\times \Sigma \;n-6\mathrm{PUFA}\right)+\left(3\;\times \;\Sigma \;n-3\mathrm{PUFA}\right)\;+\left(\Sigma \;n-3\mathrm{PUFA}/\Sigma \;n-6\mathrm{PUFA}\right)}\;HH=\frac{C18:1n-9+C18:2n-6+C20:4n-6+C18:3n-3+C20:5n-3+C22:5n-3+C22:6n-3}{C14:0\;+\;C16:0}\;PI=\left(\%\;\mathrm{dienoic}\times \;1\right)+\left(\%\;\mathrm{trienoic}\times \;2\right)+\left(\%\;\mathrm{tetraenoic}\times \;3\right)+\;+\;\left(\%\;\mathrm{hexaenoic}\times \;5\right)$$

The Folin–Ciocalteu phenol reagent (FCR), gallic acid, 2,2-diphenyl-1-picrylhydrazyl (DPPH^•^), methanol, sodium carbonate, were purchased from Sigma-Aldrich (St. Louis, MO, USA). 

Seeds of *A. cruentus* L. accessions were milled in a coffee grinder and passed through an 18 Mesh diameter sieve. Following what was previously indicated by López-Mejía et al. [35], 10 g of the obtained homogeneous seeds powder were placed in a Erlenmeyer flask, added with 150 mL of methanol and extracted, away from light, under magnetic stirring for 15 h at room temperature. Vegetal matrix was then filtered and washed trice with fresh extraction solvent, and the crude extract underwent solvent removal by rotary evaporator. Extracts were kept at 4 °C until analysis. Extraction yields (%) were calculated on the basis of seed weight and dry extract.

The DPPH^•^ assay was used to characterize the antioxidant capacity of all the obtained extracts, following the method described by Brand-Williams et al. [36]. Extract solutions in methanol over a range of concentrations from 2–10 mg/mL were prepared. To 100 µL of these solutions, 0.25 mL of 1 mM DPPH^•^ and 2 mL of methanol were added. After 20 min, the absorbance of the mixtures was read at 517 nm (Cary 60 UV-Vis spectrophotometer, Agilent Technologies, Santa Clara, CA, USA). EC_50_ values, defined as the concentration of dried extract (mg/mL solution) needed to scavenge the 50% of initial DPPH^•^, were evaluated.

The total phenols content (TPC) in the obtained extracts of each *A. cruentus* L. accession was determined according to the method developed by Karamać M. et al. [8]. The 0.25 mL of methanolic solution of the extract (5 mg/mL), 0.25 mL of Folin-Ciocalteu reagent, 0.5 mL of 10% sodium carbonate solution, and 4 mL of water were mixed. After 25 min, the absorbance was measured at 725 nm. TPC was expressed as gallic acid equivalents (GAE, mg/g) over g of seeds.

### 2.3. Statistical Analysis

A One-way ANOVA model with multiple mean comparisons, according to Tukey’s (HSD) test, was performed to determine differences between accessions, using DSAASTAT v. 1.1 software [37]. To ensure normality, percentage values were previously arcsin square root transformed; in tables, percentage data were reported.

Finally, with the aim of exploring the strength of the correlations among qualitative variables a Principal Component Analysis (PCA) of all qualitative traits (fatty acids profile, indices, TPC, DPPH^•^) was performed on the correlation matrix, due to the different measurement units, adopting the software StatistiXL (Roberts and Withers, Broadway, Nedlands, Australia). The first 2 principal components were displayed on a distance-based biplot.

For this test, we used the eigenvalue greater than 1 as component retention criteria, and, in agreement with MacCallum et al. [38], a value greater than 0.6 as acceptable scores.

## 3. Results

### 3.1. Agronomic Traits

The three studied agronomic parameters (plant height, seed yield, and thousand seed weight) showed high variability.

The eight amaranth accessions can be divided into two groYes, you can.ups in relation to plant height: The first group with an average plant height of 120 cm including accessions from the Mexico, Guatemala, Montana, and Illinois and a second group with an average plant height of 83 cm represented by Arizona, Pennsylvania, Benin, and Zaire (Table 1).

Seed yield resulted quite variable: The highest yielding accessions resulted PI 477913 and PI 538255 (0.37 kg/m^2^, on average), even though undifferentiated from all the other accessions, except PI 628793, which showed the significantly lowest seed yield (*p* < 0.05).

As well as the plant height, the thousand seed weight divided the accessions into two groups: The first group of accessions with a low thousand seed weight composed by Guatemala, Benin, and Zaire with an average value of 0.50 g, and a second group with a higher thousand seed weight composed by Mexico, Montana, PI Arizona, Pennsylvania, and Illinois with an average value of 0.80 g.

### 3.2. Oil, Fatty Acid Content, Antioxidant Activity, and Total Phenolic Content

Significant differences among the eight Amaranth accessions were observed for the oil content of seeds (Table 2). On the whole, the highest oil content was observed in the PI 606797 accession from USA, Illinois, the lowest in the PI 618962 from Benin. 

The fatty acid composition and the fatty acid classes are reported in Table 2 and Table 3, respectively. Six Saturated Fatty Acids (SFAs) were detected in amaranth seeds (Table 2); among these, palmitic acid (C16:0) was found at significant (*p* < 0.05) highest levels in PI 628793 from Zaire (Shaba), followed by the stearic acid (C18:0) in PI511717 from Guatemala (Table 2). The Myristic (C14:0), heptadecanoic (C17:0), eicosanoic (C20:0), and docosanoic (C22:0) acids were lower than 2% in all amaranth accessions (Table 2). Three MUFAs were identified and quantified (Table 3) and, among these, the most represented were oleic acid (C18:1n9) showing the significant (*p* < 0.05) highest level in PI 606797 accession from USA, Illinois, followed by the cis-11-octadecenoic acid (C18:1n7) an isomer of oleic acid, which showed the significant (*p* < 0.05) highest level in PI511717 from Guatemala (Table 2). Among PUFAs, the linoleic acid (C18:2n6) was the most represented in all the eight amaranth accessions, with a significant (*p* < 0.05) major content in PI 566896 from USA (Arizona), followed by the alpha-linolenic acid (C18:3n3) that showed the significant (*p* < 0.05) highest value in PI477913 from Mexico (Table 2).

Table 3 covers the fatty acid classes, the fatty acid ratios, and the quality indices, AI and TI. From a nutritional point of view, PI 566896 from Arizona showed the significantly (*p* < 0.05) lowest value of SFAs, and the significantly (*p* < 0.05) highest values of PUFA, n-6 PUFA and n-3 PUFA. On the contrary, PI 511717 from Guatemala showed the significant (*p* < 0.05) highest value of SFA and the lowest values of total PUFA, both n-6 and n-3 series. Consequently, the SFA/UFA ratio showed the significantly (*p* < 0.05) highest value in PI 511717 from Guatemala and the lowest in PI 566896 from Arizona, respectively. Finally, AI, TI, and H/H, strictly related to the fatty acid profile, showed the significant (*p* < 0.05) best values in PI 566896 from Arizona and the worst values in PI 511717 from Guatemala. The peroxidation index, related to the content of unsaturated fatty acids, showed the significant (*p* < 0.05) highest value in PI 566896 from Arizona.

The TPC, in the different accessions of *A. cruentus* varied significantly (*p* < 0.05) in this order: Mexico > Guatemala > Benin > Arizona > Illinois > Zaire > Montana > Pennsylvania. The IP 511717 Guatemala had no significant difference from Mexico and Benin, while it was significantly (*p* < 0.05) higher compared to the other accessions. The PI 538255 from the USA (Montana) had the same statistical value of PI 628793 from Zaire (Shaba) and the overall lowest TPC content. By considering the significativity of reported data (Table 3), PI 477913 from Mexico showed the highest (*p* < 0.05) TPC content.

Significant differences (*p* < 0.05) among the eight accessions of *A. cruentus* were observed for the antioxidant power (DPPH^•^) and the total phenol content (TPC) (Table 3).

As regards the antioxidant power, the DPPH^•^ values reached a lower (*p* < 0.05) levels in PI 628793 from Zaire (Shaba) in PI 566896 from USA, (Arizona) compared to the other accessions, excluding PI 618962 from Benin, which showed no significant difference among the accessions.

PI 628793 from Zaire (Shaba) has a low DPPH^•^ value (high antioxidant activity) despite the low amount of polyphenols because it has a high content of SFA, which is stable to oxidation (Table 3). The PI 566896 from the USA (Arizona), maintains a low DPPH^•^ value and a good TPC level despite having the highest PUFA level (Table 3); for this reason, from the health point of view, it represents the best accession. The PI 511717 from Guatemala has the highest DPPH^•^ value, despite a good amount of polyphenols, but it also has the highest SFA content (Table 3); in this case, the DPPH^•^ value found, reflects the real content of antioxidants present in the plant since SFAs are stable to oxidation.

### 3.3. Principal Component Analysis

The Principal Components Analysis was applied to identify patterns in our dataset and highlight similarities and differences among the amaranth accessions. The first two principal components account, as a whole, for 84.9% of the total data variability (Table 4). This means that only a minor quote of 15% of the variability has been lost in the simplification of the bi-plot. The first component, which explains 55.2% of the variation, was primarily a measure of C14:0, C16:0, C16:1, C18:0, C18:1 n7, C18:2 n6, C 18:3 n3, SFA, PUFA, SFA/UFA, n-3 PUFA, n-6 PUFA, IA, IT, HH, PI. Component two, responsible for 29.8% of the variation, was mainly represented by the oil, C17:0, C18:1 n9, C20:0, C22 and MUFA. Component three, responsible for 8.3% of the variation, is associated almost exclusively with TPC (Table 5).

**Figure 1:**
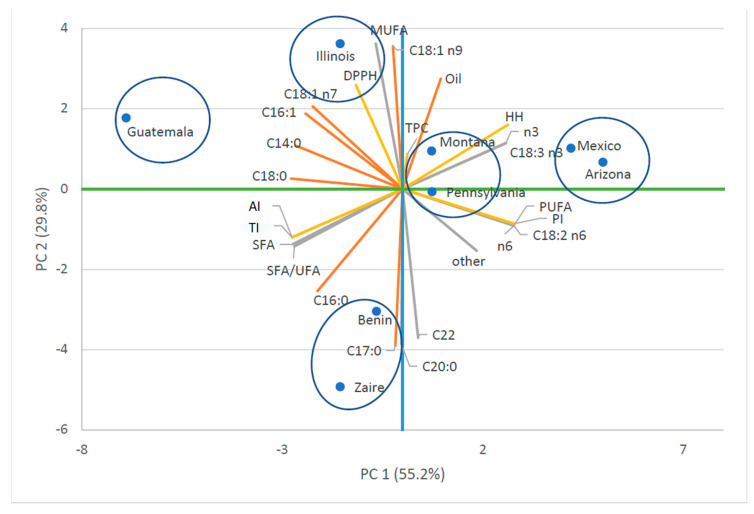
Principal component (PC) analysis of the studied amaranth accessions; SFA = Saturated fatty acids; MUFA = Monounsaturated fatty acids; PUFA = Polyunsaturated fatty acids; n3 = n-3 Polyunsaturated fatty acids; n6 = n-6 Polyunsaturated fatty acids; SFA/UFA = Saturated/Unsaturated fatty acid ratio; AI = Atherogenic Index; TI = Thrombogenic Index; HH = hypocholesterolaemic/hypercholesterolaemic ratio; PI = peroxidation index; TPC = Total Phenolic Content expressed as gallic acid equivalents (GAE mg/g seeds); DPPH^•^ radical scavenging activity was expressed as EC_50_ (concentration of dried extract mg/mL solution).

The graphical representation of the relationships between accessions and parameters showed a distinction between accessions (Figure 1). Mexico and Arizona clearly stand out for the highest values of PC1, corresponding to the accessions with higher C18:3n3, C18:2n6, PUFA, n-3 PUFA, n-6 PUFA, PI and HH ratio; Benin and Zaire had the lowest value of PC2 and a negative value of PC1 with the lowest value of oil, but a high value of SFA/UFA. Illinois and Guatemala, with a different extent, showed a negative value of PC2 and a positive value of PC1 emerging for low PUFA, but high C18.1n9, MUFA and DPPH^•^. Montana and Pennsylvania showed a lower value of PC1 showing intermediate values.

## 4. Discussion

Amaranthus genus in general, and A. cruentus, in particular, are well known to show a wide degree of polymorphism and variability [39]. This is confirmed by our data that showed high variability among A. cruentus accessions.

Plant height is an important parameter in grain crops: It must not be too short to allow a mechanical harvest (with low grain lost), but not too high, otherwise plant resource will be lost producing biomass and the plant can be easily bent by the wind, preventing combine from the harvest the grain. That said, all amaranth accessions (ranging from 78.7 to 139.7 cm) were included in an optimal interval of plant height. As a whole, tested accessions resulted shorter than how obtained by Sogbohossou and Achigan-Dako [6] on 27 A. cruentus accessions (136.9 ± 25.9 cm). Casini and La Rocca [40] found a plant height ranging from 106 to 127 cm on 10 A. cruentus accessions, while Maseko et al. [41] in a two-year study, obtained a plant height ranging from 70 to 100 cm.

Yields of tested accessions resulted higher than the data of El Gendy et al. [42] but in agreement with those reported by Casini and La Rocca [40]. Moreover, compared to results obtained on *A. cruentus* in the same cultivation environment, but with different accessions [43], all the accessions tested showed slightly higher or comparable yields, except for PI 628793 (Zaire), which gave lower grain yield. On the other hand, in a possible scenario of substitution of cereal crops traditionally adopted for animal feed, *A. cruentus* yields of PI 477913 (Mexico) and PI 538255 (Montana) resulted comparable to grain yield of barley and oat.

The thousand seed weight resulted in higher compared to that observed by Sogbohossou and Achigan-Dako [6] (0.3–0.5 g) but in agreement with Gimplinger et al. [44] (0.67 g).

It must be said that, except for PI 618962 (Benin), the highest yields seem to be associated with taller plants and the highest thousand seed weights.

*A. cruentus* seeds seem to have a higher oil content (mean 6.7%) compared to that of conventional cereals such as maize [45] or sorghum [46] with nutritional properties that are far superior to corn and wheat [47].

The oil content of the eight amaranth accessions resulted within the range reported by Grobelnik Mlakar et al. [19] (5.6– 8.1%) and within the range observed by He et al. [48] in a study conducted in Wuhan (China) with 7 genotypes of *A. cruentus* (5.57–7.41%) with different origin (Nigera, Mexico, Czechoslovakia, USA, USA, China and China). On the other hand, our values are higher than those (1.9–5.0%) observed by He et al. [49] in a second study involving 7 genotypes of *A. cruentus* with a different origin (Ghana, Taiwan Sudan, Peru, Guatemala, USA, India) and cultivated in Wuhan (China) and by El Gendy et al. [42].

Our data, according to the results observed by El Gendy et al. [42] and He et al. [48,49], underline that the genotype is able to strongly affect the oil content, as proposed by Prakash and Pale [50]. *A. cruentus* had good potential as an energy source for growing lambs and had no negative effect on the weight gain or feed utilization [51]. Bamikole et al. [52] determined the acceptable level of amaranth (unthreshed inflorescences or the seed heads of mature grain plants) as a substitute for oil cakes as a feed ingredient of concentrate diets for rabbits; they concluded that unthreshed mature grain amaranth seed heads could be used as a component of the concentrate feeds of rabbits, up to a dietary level of 10%, to partially replace the expensive oil cakes in the diets. In pig nutrition, it can be expected that the high content of lipids (particularly of essential fatty acids) may be effective in wholesome pork production as they modify the fatty acid composition of animal tissues [53]. Amaranth grain given to fatteners at a level of 25% had no significant effect on the chemical composition, physical-chemical, or sensory properties of the meat [54].

The fatty acid composition of the oil gives information about oxidative stability and nutritional quality. According to the literature [11,42,43,47,48,49], data on amaranth grain proved that palmitic acid, oleic acid, and linolenic acid were the most represented acids in the oil, whereas other fatty acids such as stearic and linolenic acid are present in the oil to a lesser extent.

The content of the most represented fatty acids, oleic, and linoleic acids, appears similar to those described by Gresta et al. [43] in seven accessions of *A. cruentus* cultivated in a Mediterranean environment. The oleic acid content found in our samples is also in agreement with those reported by Nasirpour-Tabrizi et al. [11], Jahaniaval et al. [55] and He et al. [48]. On the contrary, the linolenic acid content in our accessions was lower than that reported by Nasirpour-Tabrizi et al. [11], Jahaniaval et al. [55], and He et al. [48]. The palmitic acid level was similar to that reported by He et al. [49] but higher than that reported by Nasirpour-Tabrizi et al. [11], Gresta et al. [43], He et al. [48], and Jahaniaval et al. [55]. Moreover, although the oil content was similar, the fatty acids profile appeared very different from that reported by El Gendy et al. [42], who observed a lower level of palmitic and oleic acids and a higher level of linoleic acid.

It must be mentioned that plant oils with a good proportion of omega 3, 6, and 9 are recommended for their benefits for human and animal health. The comparison of *A. cruentus* with the main cereals used in animal feeding evidenced a lower content of linoleic acid (omega 6) and linolenic acid (omega 3), and a higher content of oleic acid (omega 9) in comparison with Maize (*Zea mays* L.) [56], wheat (*Triticum aestivum* L. and *Triticum durum* Desf.), barley (*Hordeum vulgare* L.), rye (*Secale cereale*), and triticale (*Triticosecale*) [57]. The lower levels of linoleic and linolenic acids compared to the oleic acid content could be explained by the fact that linoleic and linolenic acids are biosynthesized from the oleic acid [56]. It is worth mentioning that oleic acid is considered a heart-healthy fatty acid and is generally recommended to reduce the oxidation of LDL cholesterol, the progression of atherosclerosis, and the cardiovascular risk [58,59]. Several researchers studied the effect of the diet on the fatty acid profile in ruminants [58,59] and in the mogastric species [60] in order to increase the monounsaturated fatty acids, and particularly the oleic acid for its beneficial effects on the human and animal health. In this view, amaranth confirms its feed value for animal nutrition even if it is necessary to pay particular attention to the stakeholders using ad hoc formulations and methodology in order to exploit amaranth seeds to promote the quality of animal products.

As regards to the minor fatty acids, the stearic acid content was higher than that reported in the literature [11,42,43,48,49,55]. In addition, the linolenic acid, a fatty acid that, together with the linolenic acid, represents the essential fatty acids, was lower than that reported by El Gendy et al. [42] and by Jahaniaval et al. [55].

It must be mentioned that other authors did not find this fatty acid in the *A. cruentus* grain. [11,43,48,49]. Even a low level of linolenic acid, about 0.5%, seems to be sufficient to increase the healthy properties of this oil source [47]. In fact, as reported by Raiciu et al. [47], although amaranth has a low amount of *n*−3 PUFA, the consumption of amaranth oil leads to improve cardiovascular health, heart stimulation, regulation of the blood pressure, decrease in bad cholesterol and triglycerides from blood, and also helps to prevent metabolic imbalances of calcium and iron, which generally occur at old age.

Concerning the acidic classes, the oil of the eight accessions of *A. cruentus* was characterized by a higher content of UFAs than the SFAs, containing more than 60% unsaturated fatty acids. The levels of MUFA and PUFA were similar to those observed by El Gendy et al. [42], whereas, lower than that (71–87%) was recorded by Jahaniaval et al. [55]. On the other hand, the range of the saturated fatty acid content was higher than that recorded by El Gendy et al. [42] and by Jahaniaval et al. [55]. The ratio SFA/UFA appears significantly higher than that observed in literature by El Gendy et al. [42] (0,28), by He et al. [48] (from 0.26 to 0.32), by He et al. [49] (0.44), and by Jahaniaval et al. [55] (0.37). 

In our samples, the peroxidation index (PI) values were significantly affected by the accessions and increased with the proportion of the PUFA in the oil of seeds.

As regards to the nutritional indices, the atherogenic (AI) and thrombogenic (TI) indices, since only three SFA (C12, C14 and C16) are hypercholesterolemic, AI applied by Ulbright and Southgate [32] indicates the relationship between the sum of these three SFA and the sum of the main classes of unsaturated fatty acids, which are antiatherogenic. Therefore, AI is considered an indicator or measurement of the level of the atherogenicity and IT of the level of thrombogenicity. Kabiri et al. [61,62] observed in atherosclerotic rabbits received amaranth extract supplementation, a significant reduction of the atherogenic index.

A further beneficial effect of *Amaranthus* on health can be attributed to its hypocholesterolemic action [63]. A good approach to the nutritional evaluation of the oil should be the utilization of an index based on functional effects of fatty acids, the ratio of hypocholesterolemic: hypercholesterolaemic fatty acids (HH), computed according to the knowledge of the effects of individual fatty acids on the cholesterol metabolism [64]. This is in agreement with Plate and Areas [65] and Peiretti [66], who demonstrated that the cholesterol-lowering effect of amaranth when included in the hypercholesterolemic rabbit diet (either as oil or extruded) reduced low-density lipoprotein and total cholesterol levels in the blood. In poultry nutrition, cholesterol, triglyceride, and serum lipid peroxidation levels were lower in the broilers fed diets with 5 and 10% of Amaranth grain than control [67]. At the same time, no negative effects of the diets with various forms of amaranth on the live weight, feed conversion, carcass characteristics, and meat quality of broiler chickens [68], and health conditions, egg quality, and other eggs parameters of Lohmann Brown laying hens [69].

No references of the AI, TI, and H/H indices were found in literature in the oil from *Amaranthus* seeds.

It must be mentioned that cereals and, to a lesser extent, pseudo-cereals are an important part of diets for hypercholesterolemic patients [70]. It is well known that the bioactive components of cereals positively affect the plasma lipids in both hypercholesterolemic animals and humans. The degree of this positive influence is directly linked to the contents of the bioactive components and the antioxidant activities of cereals and pseudo-cereals [70].

Investigation on the antioxidant properties of plants is a very active field of research, and information on the phytochemicals of the various species is essential. Notwithstanding, amaranths show nutraceutical potentials, published data on the phenolic content and antioxidant properties of grain amaranth species are very limited. In this view, our results appear able to enrich scientific knowledge, also considering that the phytochemical content and antioxidant activity of crops vary with species, environmental conditions, and soil factors [71].

The comparison of our data with those of the literature was difficult since there are many assays for total antioxidant determination, and each has its limitations. According to Ou et al. [72], some antioxidant assays give different antioxidant activity trends; here we used the capacity to scavenge the “stable” free radical 2,2 diphenyl-1-picrylhydrazyl (DPPH^•^) [36]. In particular, Peiretti [66] demonstrated that in rabbit nutrition, unthreshed mature grain amaranth seed heads could be used as a component of the concentrate feeds of rabbits, up to a dietary level of 10%, and it can be considered as an effective natural antioxidant supplement capable of protecting cellular membranes against oxidative.

Concerning antioxidant activity and TPC of *A. cruentus* seeds, our data are quite consistent to those previously reported by other authors as Stănilă et al. [73], while the higher values recently found by El Gendy et al. [42] and Gresta et al. [43] can be ascribed to the different accessions or pedoclimatic conditions. The comparison of the total phenolic content of *A. cruentus* with the main cereals used in animal feeding evidenced a comparable content of TPC with oat, barley, and rice and a lower content of TPC in comparison to wheat, maize, rye, and to other pseudo-cereals like quinoa [71].

The results of the determination of the antioxidant potential have shown that, in some accessions (Mexico, Guatemala, and Benin), the antioxidant potential was higher in samples with higher TPC as described by Czerwinski et al. [70], whereas, in other accessions (Montana and Pennsylvania), to the highest antioxidant activity we observed the lowest TPC, as reported by Ou et al. [72].

As a whole, we can affirm that *A. cruentus* could be a valid substitute of the traditional cereals grain. This is also in agreement with Peiretti [66] who reported that the addition of treated amaranth grains to ruminant diets enhanced the production of microbial protein in the effluents and indicated that these grains could partially substitute barley.

PCA showed a strong similarity between some of the eight accessions and allowed us to divide them into five groups: Group 1 Mexico and Arizona, group 2 Montana and Pennsylvania, group 3 Benin and Zaire, group 4 Illinois, and group 5 Guatemala. Moreover, from a nutritional point of view, the PCA shows that the combination of the higher values of C18:3n3, C18:2n6, PUFA, n-3 PUFA, n-6 PUFA, TPC and HH ratio, and the lower values of SFA, IA, IT and SFA/UFA ratio were detected in Mexico and Arizona accessions, strengthening the reliability of the Quality Indices proposed by Ulbricht and Southgate [32]. These authors suggested that the AI and TI, strictly related to the entire fatty acid profile, might better characterize the health benefits of a vegetable or animal food than a simple approach based on fatty acid classes or fatty acid ratios [74,75]. Concerning the positive effect in the food chain “from feed to food,” Mexico and Arizona seem to be the best accessions to obtain a healthy ingredient for animal feed. Interestingly, the Illinois accession comes out for the highest levels of C18:1n9 and MUFA.

## 5. Conclusions

The first consideration of the results emerging in our trial is that grain yield of *A. cruentus* showed values not far from those of many cereals such as barley and oat traditionally used for animal feed.

From a qualitative point of view, *A. cruentus* grain showed higher lipid content than most cereal grains and thus can be classified among the plants in which oil is rich in n-6 PUFA along with hemp (*Cannabis sativa*) and safflower (*Carthamus tinctorius*).

The studied seed-oil composition revealed a high content of essential fatty acid n-6 (linoleic acid) and n-9 (oleic acid). Therefore, this seed oil can be used to balance the diet according to animal species requirements (diet, supplements, biofunctional compounds), by including the appropriate amount of *A. cruentus* in feed to enrich in fatty acids the products of animal origin.

Moreover, *A. cruentus* showed a comparable total phenolic content with oat, barley, and rice and, therefore, could be a valuable substitute for traditional cereals in animal feeding.

In this light, we can say that there is a good opportunity for the enhancement of important parameters to use it in animal nutrition as a bioactive component source.

In order to make the most of the bioactivity and health effects of *A. cruentus* seeds, further researches need to be carried out focusing on ad hoc formulations in relation to the animal species, according to the different metabolic pathway of unsaturated fatty acids in ruminants and monogastrics.

Among amaranth accessions, Mexico and Arizona emerged as the best healthy ingredient for animal feeding.

## Figures and Tables

**Table 1:** Main agronomic traits of the studied amaranth cruentus accessions.

Accession	Origin	Plant Height (cm)	Seed Yield (kg/m^2^)	1000 Seed Weight (g)
PI 477913	Mexico	129.3 ^a^	0.37 ^a^	0.84 ^a^
PI 511717	Guatemala	139.7 ^a^	0.27 ^ab^	0.59 ^b^
PI 538255	USA, Montana	123.7 ^a^	0.37 ^a^	0.84 ^a^
PI 566896	USA, Arizona	81.0 ^b^	0.22 ^ab^	0.75 ^a^
PI 605354	USA, Pennsylvania	84.0 ^b^	0.23 ^ab^	0.71 ^a^
PI 606797	USA, Illinois	127.0 ^a^	0.31 ^a^	0.85 ^a^
PI 618962	Benin	78.7 ^b^	0.25 ^ab^	0.49 ^b^
PI 628793	Zaire, Shaba	85.0 ^b^	0.09 ^b^	0.43 ^b^
Average		106.0	0.27	0.69

PI = Plant Inventory. Mean values (a, b) with different letters within the same column differ significantly (*p* < 0.05).

**Table 2:** Mean values of three replications of oil content (g/100g, as fed) and fatty acid profile (g/100 g) * in the seven varieties amaranthus seeds.

Accession	Origin	OIL	C14:0	C16:0	C16:1	C17:0	C18:0	C18:1 n9	C18:1 n7	C18:2 n6	C18:3 n3	C20:0	C22	Other
PI 477913	Mexico	7.05 ^ab^	0.42 ^f^	25.78 ^g^	0.24 ^d^	0.48 ^b^	4.44 ^h^	32.35 ^e^	1.41 ^cd^	30.83 ^b^	0.47 ^a^	0.74 ^e^	0.38 ^d^	2.50 ^a^
PI 511717	Guatemala	5.77 ^c^	1.84 ^a^	29.05 ^c^	1.00 ^a^	0.44 ^b^	11.11 ^a^	32.93 ^d^	2.16 ^a^	18.90 ^h^	0.29 ^e^	0.67 ^f^	0.29 ^f^	1.32 ^d^
PI 538255	USA, Montana	7.12 ^ab^	0.70 ^cd^	27.50 ^d^	0.26 ^cd^	0.47 ^b^	5.23 ^f^	34.42 ^b^	1.48 ^c^	26.57 ^e^	0.36 ^bc^	0.84 ^d^	0.40 ^cd^	1.77 ^bc^
PI 566896	USA, Arizona	6.09 ^bc^	0.39 ^f^	24.20 ^h^	0.23 ^d^	0.44 ^b^	4.99 ^g^	31.02 ^f^	1.42 ^c^	34.03 ^a^	0.45 ^a^	0.76 ^e^	0.35 ^de^	1.72 ^c^
PI 605354	USA, Pennsylvania	5.97 ^bc^	0.60 ^e^	27.20 ^e^	0.25 ^d^	0.47 ^b^	5.72 ^e^	33.58 ^c^	1.28 ^d^	27.40 ^d^	0.34 ^cd^	0.92 ^c^	0.44 ^bc^	1.81 ^bc^
PI 606797	USA, Illinois	7.29 ^a^	0.91 ^b^	26.78 ^f^	0.71 ^b^	0.36 ^c^	7.97 ^b^	36.03 ^a^	1.83 ^b^	22.75 ^g^	0.39 ^b^	0.64 ^f^	0.32 ^ef^	1.33 ^d^
PI 618962	Benin	5.59 ^c^	0.78 ^c^	29.46 ^b^	0.33 ^c^	0.65 ^a^	7.12 ^d^	28.58 ^g^	1.43 ^c^	28.04 ^c^	0.35 ^c^	1.06 ^b^	0.48 ^b^	1.72 ^c^
PI 628793	Zaire, Shaba	5.64 ^c^	0.64 ^de^	30.79 ^a^	0.28 ^cd^	0.70 ^a^	7.44 ^c^	28.53 ^g^	1.41 ^cd^	26.08 ^f^	0.32 ^d^	1.24 ^a^	0.56 ^a^	2.03 ^b^
Average		6.31	0.78	27.79	0.41	0.50	6.75	32.18	1.55	26.82	0.37	0.86	0.40	1.77

PI = Plant Inventory. Mean values with different letters (a–g) within the same column differ significantly (*p* < 0.05); * The concentration of individual fatty acids was expressed per total fatty acid methyl esters identified; C14:0 = myristic acid. C16:0 = palmitic acid. C16:1 = palmitoleic acid. C17:0 = heptadecanoic acid. C18:0 = stearic acid. C18:1n9 = oleic acid. C18:1n7 = cis-vaccenic acid. C18:2n6: linoleic acid. C18:3n3 = alfa-linolenic acid. C20:0 = eicosanoic acid. C22:0 = docosanoic acid.

**Table 3:** Mean values of three replications of fatty acid classes (g/100 g) *, ratios, quality indices, and antioxidant properties in the seven varieties of amaranthus seed.

Accession	Origin	SFA	MUFA	PUFA	SFA/UFA	n3	n6	AI	TI	HH	PI	TPC	DPPH
PI 477913	Mexico	32.21 ^f^	33.99 ^d^	31.30 ^b^	0.49 ^f^	0.47 ^a^	30.83 ^b^	0.42 ^f^	0.90 ^f^	2.43 ^b^	31.77 ^b^	0.36 ^a^	0.459 ^a^
PI 511717	Guatemala	43.40 ^a^	36.09 ^b^	19.18 ^h^	0.79 ^a^	0.29 ^e^	18.90 ^h^	0.66 ^a^	1.48 ^a^	1.69 ^g^	19.47 ^h^	0.34 ^ab^	0.495 ^a^
PI 538255	USA, Montana	35.13 ^e^	36.17 ^b^	26.93 ^e^	0.56 ^e^	0.36 ^bc^	26.57 ^e^	0.48 ^e^	1.03 ^e^	2.18 ^cd^	27.29 ^e^	0.17 ^e^	0.455 ^a^
PI 566896	USA, Arizona	31.14 ^g^	32.67 ^e^	34.48 ^a^	0.46 ^g^	0.45 ^a^	34.03 ^a^	0.38 ^g^	0.85 ^g^	2.66 ^a^	34.93 ^a^	0.31 ^c^	0.329 ^b^
PI 605354	USA, Pennsylvania	35.35 ^e^	35.11 ^c^	27.74 ^d^	0.56 ^e^	0.34 ^cd^	27.40 ^d^	0.47 ^e^	1.04 ^e^	2.21 ^c^	28.08 ^d^	0.14 ^f^	0.481 ^a^
PI 606797	USA, Illinois	36.98 ^d^	38.56 ^a^	23.13 ^g^	0.60 ^d^	0.39 ^b^	22.75 ^g^	0.49 ^d^	1.12 ^d^	2.14 ^d^	23.52 ^g^	0.25 ^d^	0.475 ^a^
PI 618962	Benin	39.55 ^c^	30.34 ^f^	28.39 ^c^	0.67 ^c^	0.35 ^c^	28.04 ^c^	0.55 ^c^	1.23 ^c^	1.88 ^e^	28.74 ^c^	0.33 ^b^	0.396 ^ab^
PI 628793	Zaire, Shaba	41.36 ^b^	30.21 ^f^	26.40 ^f^	0.73 ^b^	0.32 ^d^	26.08 ^f^	0.59 ^b^	1.33 ^b^	1.75 ^f^	26.73 ^f^	0.17 ^e^	0.303 ^b^
Average		36.89	34.14	27.19	0.61	0.37	26.82	0.51	1.12	2.12	27.57	0.26	0.424

PI = Plant Inventory. Mean values with different letters (a–g) within the same column differ significantly (*p* < 0.05); * The concentration of fatty acids was expressed per total fatty acid methyl esters identified; SFA = Saturated fatty acids; MUFA = Monounsaturated fatty acids; PUFA = Polyunsaturated fatty acids; n3 = n-3 Polyunsaturated fatty acids; n6 = n-6 Polyunsaturated fatty acids; SFA/UFA = Saturate/Unsaturated fatty acid ratio; AI = Atherogenic Index; TI = Thrombogenic Index; HH = hypocholesterolaemic/hypercholesterolaemic ratio; PI = peroxidation index; TPC = Total Phenolic Content expressed as gallic acid equivalents (GAE mg/g seeds); DPPH^•^ radical scavenging activity was expressed as EC_50_ (concentration of dried extract mg/mL solution).

**Table 4:** Explained Variance (Eigenvalues) of the PCA.

Value	PC 1	PC 2	PC 3
Eigenvalue	13.791	7.442	2.068
% of Var.	55.163	29.769	8.272
Cum.%	55.163	84.932	93.203

Principal component (PC) analysis.

**Table 5:** Component loadings of the PCA.

Variable	PC 1	PC 2	PC 3
Oil	0.326	0.692	0.321
C14:0	−0.915	0.272	−0.235
C16:0	−0.726	−0.640	0.146
C16:1	−0.827	0.473	−0.279
C17:0	−0.061	−0.981	−0.057
C18:0	−0.952	0.066	−0.254
C18:1 n9	−0.080	0.894	0.439
C18:1 n7	−0.766	0.517	−0.334
C18:2 n6	0.952	−0.229	−0.153
C18:3 n3	0.881	0.288	−0.271
C20:0	−0.010	−0.971	0.224
C22	0.132	−0.931	0.311
Other	0.634	−0.388	0.029
SFA	−0.934	−0.345	−0.065
MUFA	−0.230	0.910	0.339
PUFA	0.954	−0.223	−0.155
SFA/UFA	−0.924	−0.360	−0.104
n3	0.881	0.288	−0.271
n6	0.952	−0.229	−0.153
AI	−0.943	−0.303	−0.091
TI	−0.944	−0.303	−0.111
HH	0.899	0.401	−0.051
PI	0.955	−0.217	−0.157
TPC	0.042	0.219	−0.922
DPPH^•^	−0.398	0.652	0.209

Principal component (PC) analysis; SFA = Saturated fatty acids; MUFA = Monounsaturated fatty acids; PUFA = Polyunsaturated fatty acids; n3 = n-3 Polyunsaturated fatty acids; n6 = n-6 Polyunsaturated fatty acids; SFA/UFA = Saturate/Unsaturated fatty acid ratio; AI = Atherogenic Index; TI = Thrombogenic Index; HH = hypocholesterolaemic/hypercholesterolaemic ratio; PI = peroxidation index; TPC = Total Phenolic Content expressed as gallic acid equivalents (GAE mg/g seeds); DPPH^•^ radical scavenging activity was expressed as EC_50_ (concentration of dried extract mg/mL solution).
